# Sealing and unsealing effect of filamentous fungi in CEM I cement mortars with polycarboxylic admixture

**DOI:** 10.1371/journal.pone.0299221

**Published:** 2024-02-23

**Authors:** Elżbieta Stanaszek-Tomal

**Affiliations:** Chair of Building Materials Engineering, Faculty of Civil Engineering, PK Cracow University of Technology, Cracow, Poland; Mirpur University of Science and Technology, PAKISTAN

## Abstract

Biodeterioration may take place due to the influence of microorganisms, leading to the deterioration or destruction of materials. This process involves distinct mechanisms of material breakdown, such as mechanical damage, decomposition, corrosion induced by microorganisms, and fouling caused by living organisms. Research and literature data on biological corrosion of polymer-modified cement materials (polycarboxylate resin) are ambiguous and even contradictory. Samples, 20x20x160 mm in size, made of cement-polymer mortar with 5% polycarboxylate resin were prepared for the test, with 6 specimens for each determination. The study determined the level of contamination of the material, using the culture method and COD, after 18 months of exposure to *Penicillium* and *Cladosporium* fungi. In addition to this, bulk moisture content, water absorption, flexural strength and internal structure were determined using a scanning microscope and EDS analysis was carried out. The beneficial role of polymers has been demonstrated, e.g. by sealing the structure of cement-polymer materials. However, there is also an increased development of biocorrosion associated with organic material as a food source, especially for molds that colonize materials in favorable conditions. The test results showed that there was a strong growth of mold fungi on the material and the mass humidity was high. However, the strength increased slightly, and after further exposure to a corrosive environment it decreased again.

## 1. Introduction

Polymer additives for cementitious plastics have gained a permanent position [1] among modern construction materials. Superplasticisers are of particular importance in concrete technology, with polycarboxyl resins being used in high strength concretes (BWW) self-compacting concretes (SCC) [2] and in mixtures for underwater concreting. Polymer cement concretes (PCC) are obtained by adding a suitably selected polymer to the slurry. In such a material, the polymer forms a separate phase, interacting with the cement binder. The effect of the modifier may be either physical-chemical (pre-mix modifiers, i.e. modifiers that are added to the concrete mixture in a polymerised form and do not undergo further transformations) or chemical—the polymer then acts as an additional binder (post-mix modifiers, i.e. modifiers that polymerise after being added to the concrete admixture in parallel with the setting of the cement binder). The most commonly used polymer additives include acrylic and epoxy resins and synthetic rubber latexes. Concrete or cement-polymer mortars were developed as a response to damage to cementitious materials. Their role is to protect and repair concrete structures, but also to bond and are used in shotcrete (SPCC) [3, 4]. Many studies have shown that cement-polymer mortars have increased adhesion with a reduction in permeability and porosity [5, 6]. Results have also shown that the presence of the polymer hinders the penetration of aggressive agents into the mortar, resulting in increased resistance to both carbonation [7] and chloride [8] ion diffusion, and as well as frost resistance (freeze-thaw) [9]. During their service life, polymer-modified mortars can be exposed to a variety of micro-organism species. The environment-material interaction can result in changes to the surface and functional properties of the material [10]. As a result of their action, biodeterioration may occur, i.e. the destruction of material as a result of the action of microorganisms. In biodeterioration, there are several destruction mechanisms, such as mechanical, decomposition, corrosion induced by microorganisms and fouling by living organisms. However, in the case of mineral technical materials, corrosion caused by microorganisms and surface fouling will occur. The question is what mechanisms will occur in the case of cement-polymer mortars/concretes. Will there be another mechanism in this case, i.e. decomposition? Ongoing research and literature data on microbe-stimulated corrosion of materials with polymer modifiers are inconclusive and even contradictory [11, 12]. The beneficial role of polymers has been demonstrated, mainly by sealing the structure of cementitious materials [13], as well as the increased development of biocorrosion associated with organic material as a source of food, especially for moulds that colonise materials in favourable conditions (high humidity, temperature). It therefore becomes important to answer the question of whether and to what extent concretes and mortars containing a polycarboxyl superplasticiser are susceptible to the development of microorganisms and what is their effect on the durability of concrete composites.

## 2. Materials and methods

### 2.1. Polymer-cement mortars

The composition of the cement-polymer mortar is given in Table 1.

**Table 1:** Composition of the tested composite.

	Designation	Content [g]			
		Cement	Sand	Water	Polymer Modifier
CEM I mortar modified with polycarboxylate	CMPK	100	300	46.75	5

Mortar samples were prepared using quartz sand as per requirement.

The ratio of w/c in the tested mortar was 0.5. A test piece of 20x20x160 mm was made from the mortar. After preparation, they were compacted on a shaker through 30 shakes to remove air from the mortars. The test pieces prepared in the mould were stored under foil for 24 hours. After removal from the mould, the specimens were then cured under film for a further 2 days and then at +20°C ± 2°C and 60% ± 5% relative humidity as per standard procedure [14]. The total conditioning time of the samples was 28 days.

### 2.2. Corrosive environment

Pure cultures of *Penicillium chrysogenum* (abbreviated P.ch.) (LOCK 0531, streina F00680) and *Cladosporium herbarum* (abbreviated C.h.) (LOCK 0490, streina E123) from the collection of pure cultures of microorganisms (LOCK) from the Institute of Fermentation Technology and Microbiology in Łódź, Poland, were used for the study. Pure cultures of filamentous fungi were transferred into 10 ml of distilled water. Petri dishes were inoculated with the suspension created from the selected fungal culture and placed at 25°C and 95% relative humidity for five days. After this time, a clean and sterile swab was taken into 10 ml of distilled water so that the suspension had a spore count of 1 × 10^6^ spores per ml). The suspension thus prepared was used to contaminate previously prepared samples of the cement-polymer composite.

### 2.3. Temperature and humidity parameters

The contaminated test items were placed in a biological test chamber at 25°C and 95% relative humidity.

### 2.4. Experimental time

Tests were conducted at 6, 12 and 18 months after contamination with filamentous fungi. Results at 6 and 12 months are presented in the publication [15]. They are used to show the variability of the tested diagnostic characteristics over time.

Reference samples, i.e. materials not contaminated with filamentous fungi and a cement material without admixture applied, were used for comparison.

### 2.5. Diagnostic features

The diagnostic features of the processes taking place were, firstly, indicators relating to the development of micro-organisms in the mortars and, secondly, parameters representing the effect on the properties of the plastics. Changes were determined by:

- the number of colony-forming units of fungi cfu, counted per 100 cm^2^ of the surface area of the tested plastic. The counting of microorganisms using an indirect method consists of spreading a suitably diluted suspension of microorganisms on a solidified medium, also known as solid medium. The grown colonies are then incubated and lysed. A very important step is to prepare the correct dilution of the suspension, i.e. so that there are 30–300 cells in 1 cm^3^, as this number of colonies is the easiest to count on the plate. A lower number of colonies increases the error and a higher number makes counting more difficult. A series of dilutions from two separately analysed samples is prepared in parallel. The result of the determination is given in the unit [cfu/100 cm^2^],
- Chemical Oxygen Demand Index (COD). It shows the oxygen demand used for the oxidation of organic and inorganic compounds in the analysed materials. The COD determination was carried out using an AQUALYTIC AL450 photometer at 480 nm and an AL125 reactor. The method was based on the determination of the loss of dichromate concentration after two hours of oxidation in the presence of potassium dichromate, sulphuric acid and silver sulphate (as catalyst) at 148°C. Reaction tubes containing Macherey-Nagel reagent, dedicated to a specific photometric device, were used for the test. The result is given in the unit [mgO_2_/dm^3^].
- mass moisture content (μ_M_) and absorbability. These were determined in accordance with PN¬-85/B-04500 [279]. Bulk moisture content and absorbability were tested on samples made of both contaminated and uncontaminated plastics with dimensions of 20 x 20 x 160 mm by determining the mass and absorption of water. The test materials were weighed and then dried at 105°C to constant weight. The mass moisture content is calculated using the formula: μm = (m_w_-m_s_)100%/m_s_, where: m_w_—mass of wet sample [g], m_s_—mass of dry sample [g]. The absorbability test was carried out in accordance with ASTM standard procedure [16]. Samples were dried at 105°C in an oven until the difference in mass between two measurements at 24-hour intervals was less than 0.5%. After cooling, the samples were immersed in water at approximately 21°C for 48 hours. The absorbability is calculated using the formula: nm = (m_n_-m_s_)100%/m_s_, where: m_n_—mass of sample saturated with water [g]; m_s_—mass of dry sample [g].
- for the determination of flexural strength (f_t_), ZWICK Z100 testing machine of 0.5 accuracy class was used, with a three-point static scheme as shown. The test beam was placed on two lower supports (rollers) with a span of 120 mm, and a concentrated force was applied at the centre of the span [15]. Each time the test was performed, the cross-sectional dimensions of the specimen were measured at the point of breakage to the nearest 0.01 mm. The results obtained were evaluated in accordance with DIN EN 1015–11:2001 / A1:2007 [17].
- the structure, which was studied with a Zeiss EVO MA10 scanning electron microscope equipped with a Bruker XFlash 6/30 energy-dispersive X-ray spectrometer. Observations of microstructure changes and the presence of fungi were carried out on the unsprayed surfaces of the test samples. In addition, quantitative spot and surface analysis of elements was performed using EDS at selected locations.

The table and figures show the average values of the results from the 6 determinations. The scatter of the results did not exceed 0.1%.

## 3. Results

The results obtained for the development of micro-organisms in the mortars after 6, 12 and 18 months of fungal contamination are given in Table 2

**Table 2:** Contamination results expressed in different parameters for the fungal *species Penicillium chrysogenum* and *Cladosporium herbarum*. Results from 6 and 12 months published in [15].

Parameter	Time [month]			
	0	6	12	18
	***Penicillium chrysogenum***			
Contamination [cfu/100cm^2^]	0.00E+00	6.27E+07	7.30E+07	7.65E+07
COD [mgO2/dm^3^]	21.50	47.06	66.63	75.36
	***Cladosporium herbarum***			
Contamination [cfu/cm^2^]	0.00E+00	6.78E+07	7.34E+07	7.72E+07
COD [mgO2/dm^3^]	21.50	51.38	76.22	85.91

**Fig 1:**
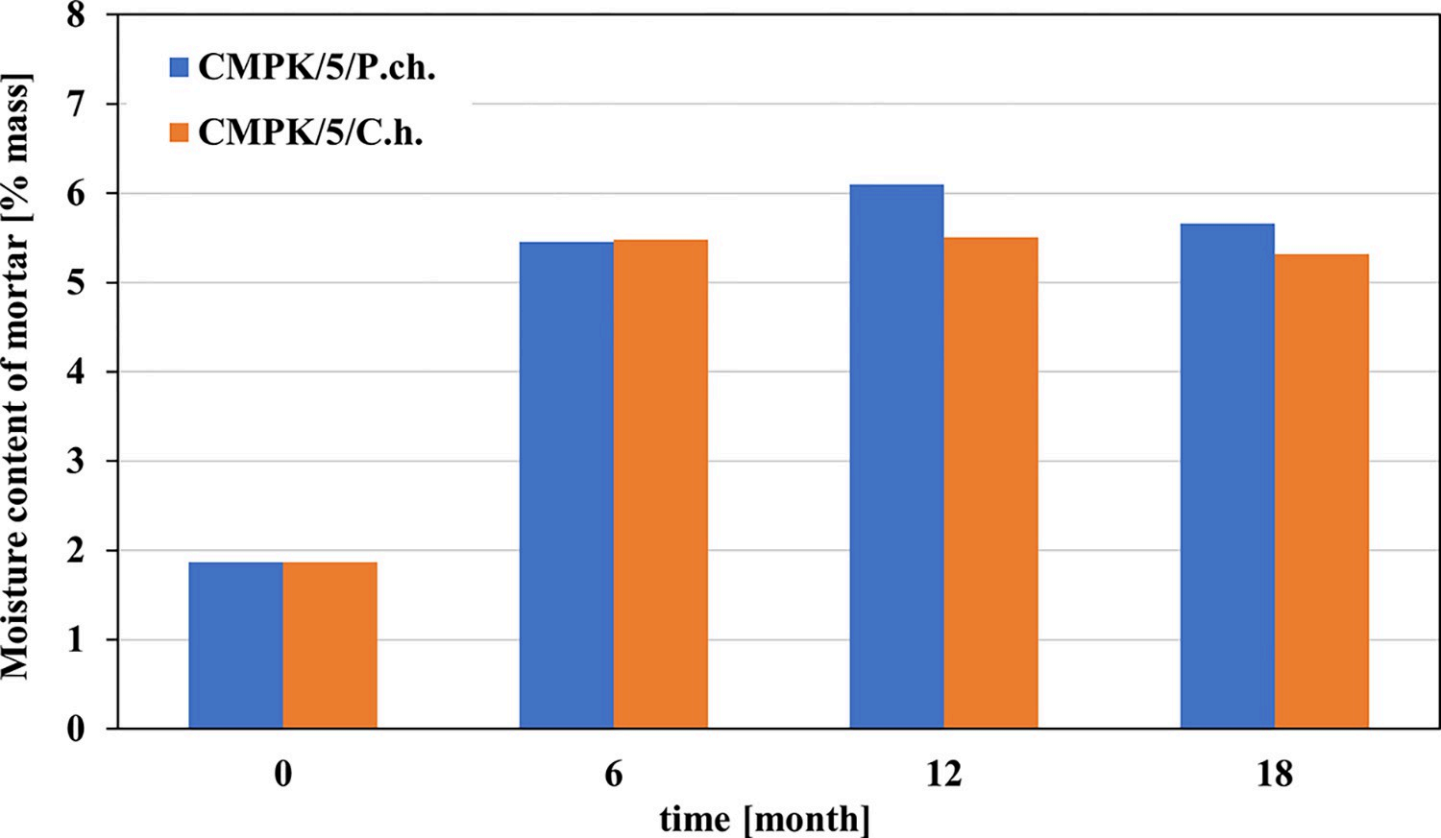
Changes in mass moisture content of a cement-polymer composite doped with 5% polycarboxylate (CMPK) caused by the development of the filamentous fungi *Penicillium chrysogenum* (blue) and *Cladosporium herbarum* (orange). Results from 6 and 12 months published in [15].

**Fig 2:**
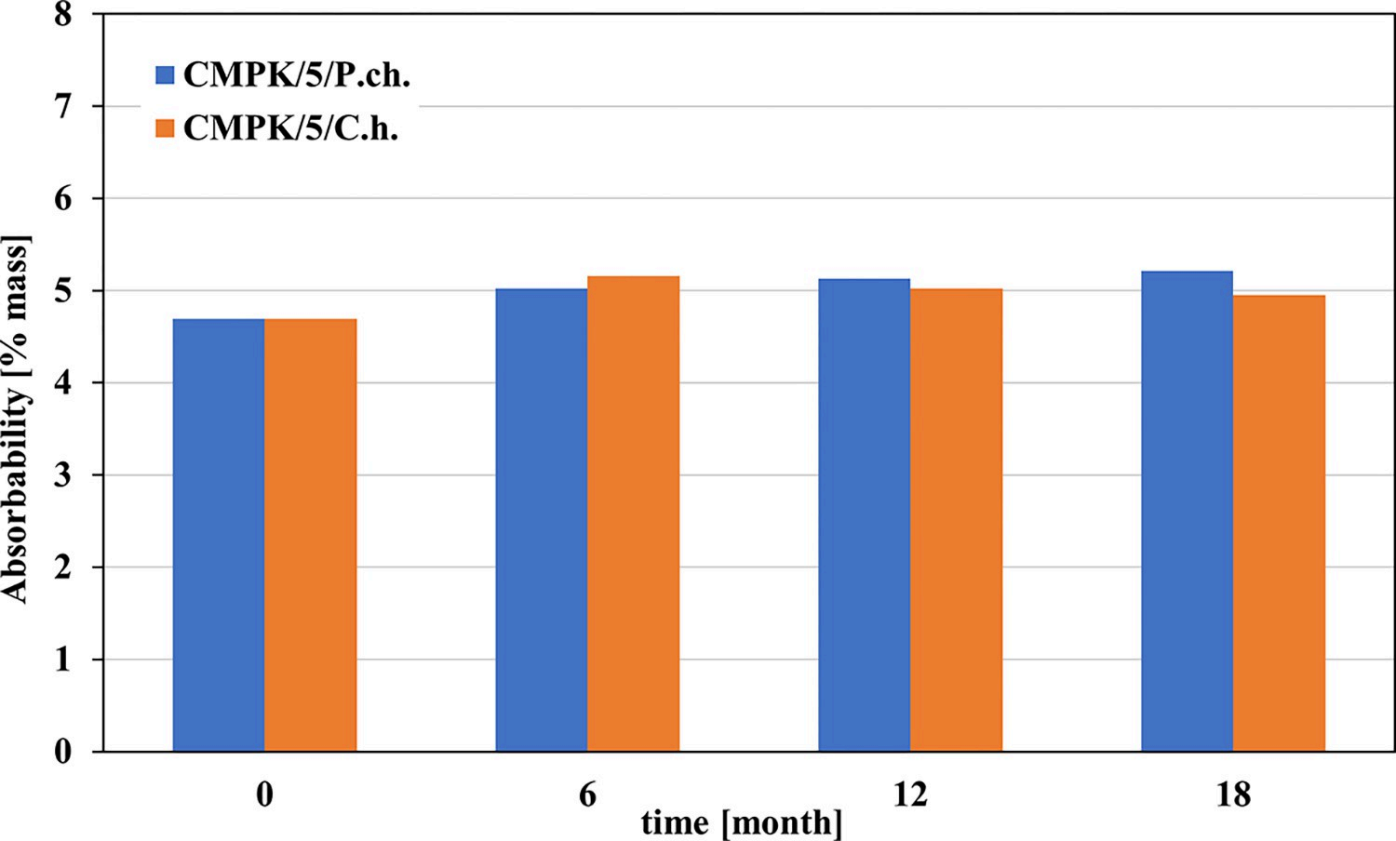
Changes in absorbability of a cement-polymer composite doped with 5% polycarboxylate (CMPK) caused by the development of the filamentous fungi *Penicillium chrysogenum* (blue) and *Cladosporium herbarum* (orange). Results from 6 and 12 months published in [15].

In contrast, the effect on water absorption capacity is illustrated in Figs 1 and 2

**Fig 3:**
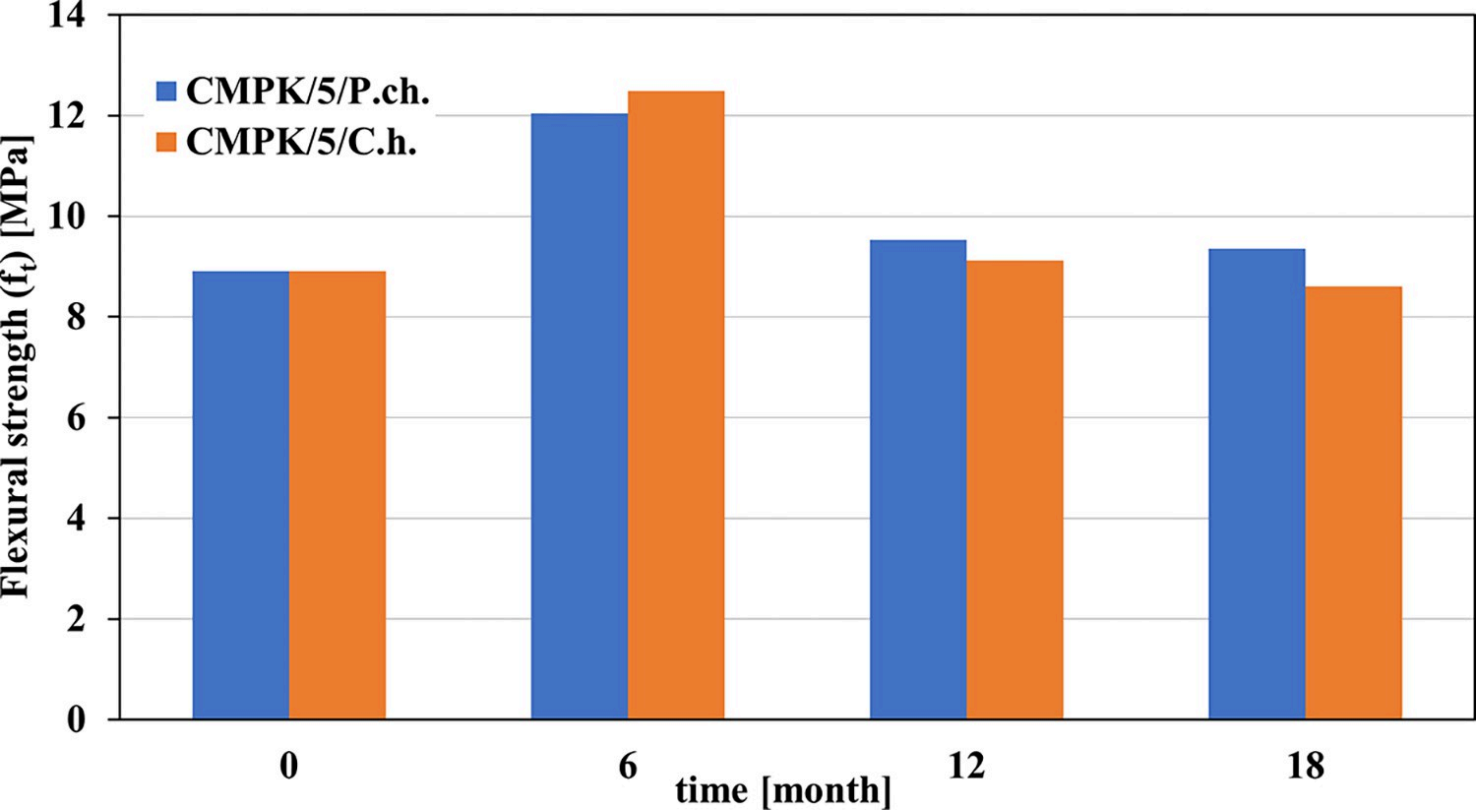
**Changes in the flexural strength (f**_**t**_**) of a cement-polymer composite with an admixture of 5% polycarboxylate (CMPK) caused by the growth of filamentous fungi *Penicillium chrysogenum* (blue) and *Cladosporium herbarum* (orange).** Results from 6 and 12 months published in [15].

Fig 3 shows the changes in flexural strength of cement-polymer mortars exposed to a biological environment in the form of filamentous fungi.

**Fig 4:**
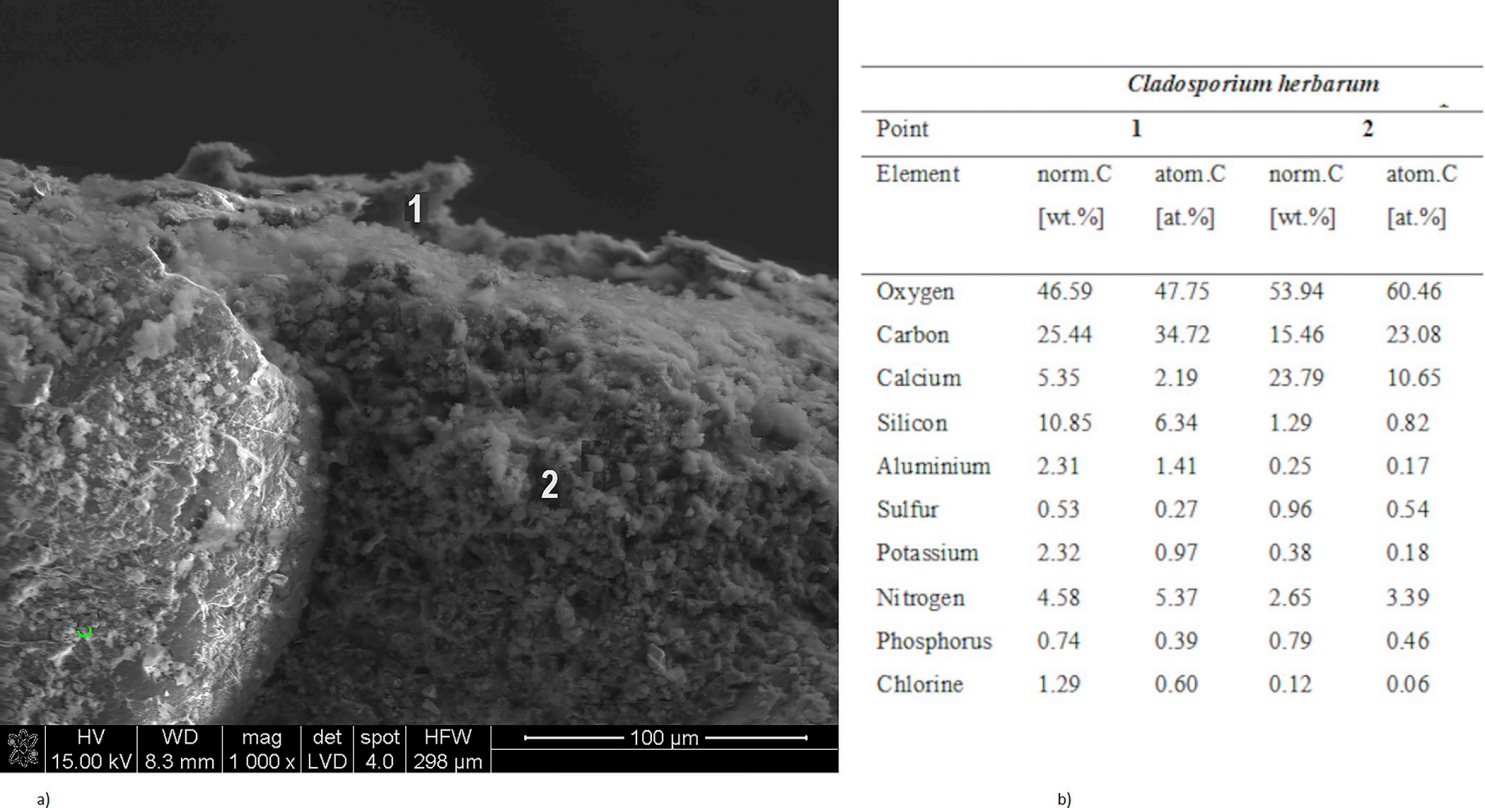
(a) Microstructure of CMPK mortar treated with *Cladosporium herbarum*, after 18 months of exposure (b) analysis from the surface of points 1 and 2 in Fig 4A.

**Fig 5:**
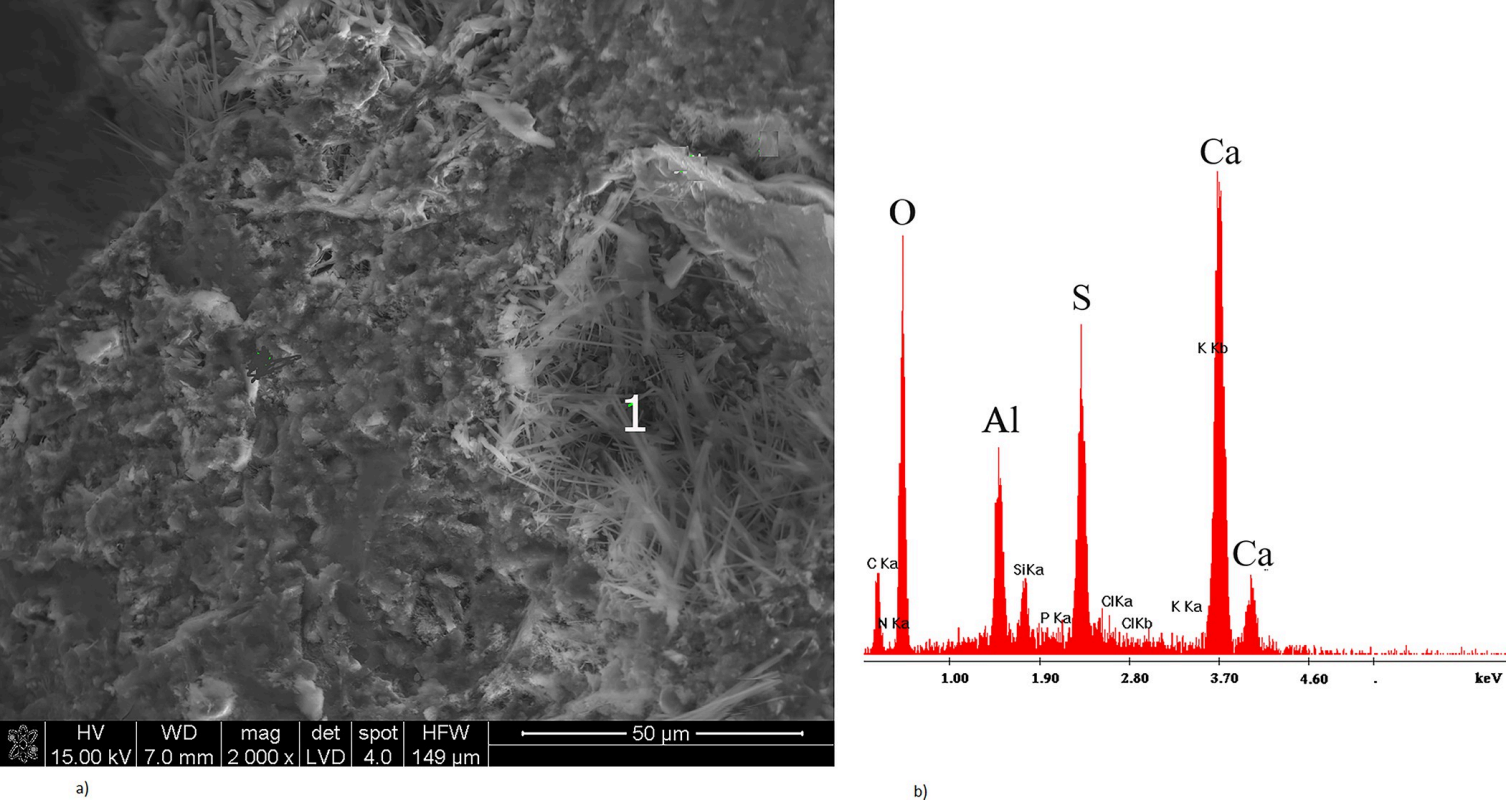
(a) Microstructure of CMPK mortar treated with *Penicillium chrysogenum* after 18 months of exposure with visible gypsum; (b) analysis from the surface of points 1 in Fig 5A.

Figs 4A and 5A show the corrosion products of mortars at 1000–2000× magnification, while Figs 4B and 5B show the EDS analysis at selected points.

## 4. Discussion

The obtained CFU results (Table 2) show strong growth of fungi of both the *Penicillium* and *Cladosporium* species. However, the number of colony-forming units is slightly higher for *Cladosporium herbarum* contamination.

COD tests (see Table 2) illustrate the demand for oxygen used for the oxidation of organic and inorganic compounds in the tested materials. In mortars without organic modifiers, only inorganic compounds are oxidized. It can therefore be assumed that the increase in COD for modified mortars is related to the oxidation of polymer modifiers. However, the growth in cement-polymer mortars contaminated with microorganisms is related to the oxidation of organic matter originating from microorganisms. Uncontaminated modified mortars also use oxygen to oxidize organic modifiers. After 18 months of fungal growth in mortars with polycarboxylic resin, the oxygen demand increases approximately three times compared to uncontaminated material. This indicator shows the content of microorganisms in the tested materials, but also the change in the amount of polymer additive.

As a result of contamination with filamentous fungi, the moisture content of CMPK mortars increases approximately 2.5 times after 6 months. For *Cladosporium* it remains practically at a similar level for the following months, while for *Penicillium* it increases after the next 6 months and decreases after 18 months. Contamination causes a strong increase in water absorption compared to reference samples. However, in relation to humidity, it shows lower values for both species of fungi and at all periods of exposure to the microbiological environment.

The changes in the bending strength of polymer cement materials as a result of the action of fungi presented in Fig 3 indicate opposite changes. After the first 6 months of exposure to microorganisms, the flexural strength increases by approximately 35 and 40% for *Penicillium chrysogenum* and *Cladosporium herbarum*, respectively. After another 6 months, it decreases by 21 and 27% to remain at a similar level for the following months. The increase in strength may be related to the deposition of corrosion products in the pores and their mineralization with CaCO_3_. Results are presented in [18] in which the sealing of the structure was noticeable due to the formation of calcium carbonate. However, in these tests, 3% polycarboxylate admixture was used. However, lowering the exposure in the following months causes the structure to become untight. Fungi, like other microorganisms, cause the formation of calcium carbonate. Microbially induced carbonate precipitation (MICP) is a mechanism by which the formation of calcium carbonate is induced by microbial activity. This mechanism is used to self-heal cracks in concrete [19]. However, in the present study, the amount of calcium carbonate decreased over time, although the fungi of both species continued to develop (high CFU/100 cm^2^ and COD).

The contaminated mortars showed a significant content of calcium carbonate, which was located in the pores and microcracks of cement-polymer materials with an admixture of 5% polycarboxylate. The EDS analysis also revealed the presence of secondary ettringite crystallizing in the leaks (Fig 5A and 5B). The CaCO_3_ (Fig 4A and 4B) and 3CaO∙Al_2_O_3_·3CaSO_4_·32H_2_O crystals were observed both with *Penicillium chrysogenum* and *Cladosporium herbarum* contamination. The results presented in Fig 4A and 4B present the surface analysis of the tested samples contaminated with *Cladosporium herbarum* after 18 months of exposure to a corrosive environment. At point no. 1 in Fig 4, a mycelium hypha overgrown with calcium carbonate and partially with the CSH phase is visible, which is confirmed by the content of C, O, Ca and Si elements. In point two of the same drawing, calcium carbonate is visible and confirmed by Fig 4B.

## 5. Conclusion

The surfaces of most materials are naturally porous, especially the layer troweled from above, therefore the texture of the surface of stone, concrete and mortar may increase moisture. The rough surface concentrates moisture in micro-cracks or pores. They are usually where the greatest growth of microorganisms occurs [20]. Moisture comes from atmospheric conditions, but primarily from the metabolic activity of microorganisms. The ambient moisture is 1–2.5% by mass. Everything else is produced by microorganisms. Depending on the type of microorganisms or complex environments, it ranged from about 5 to 6% by mass. This indicates that the metabolic processes of microorganisms are functioning efficiently.

The strength of cement-polycarboxylic materials tends to seal the structure and then to release it, ultimately returning the tightness to its initial state, similar to that of the reference materials.

As a result of the action of filamentous fungi of the genus Penicillium and Cladosporium, two opposing processes occur in cement-polymer composites, related to the deposition of sulphate corrosion products (ettringite, gypsum) [18] and calcium carbonate (the result of mineralization of organic substances, including polymer modifiers in the microcracks. cement mortars and, on the other hand, unsealing of the structure (microcracks), as well as increasing porosity. These processes occur one after another.

Perhaps carbonate corrosion of mortars occurs during exposure to mold fungi. This is possible because fungi that emit carbon dioxide are still growing and there is high humidity both in the air and due to the presence of fungi. As a result, calcium bicarbonate may be produced. It is easily soluble and washes out of the material. It is true that the author did not observe large amounts of white coating on the materials. At the moment, this is just a concept that needs to be tested and will be a further direction of research.

The research carried out on the influence of mold fungi on cement materials modified with polycarboxylic resin showed that these resins, used in concrete technology as superplasticizers, tighten and slightly unseal the already sealed initial structure of cement mortars, which reduces the susceptibility of these materials to the action of microorganisms. Unfavorable changes in humidity, water absorption and COD prove that these mortars are also, although to a lesser extent, subject to biodeterioration, the estimated rate of which is four times lower compared to polymer-free materials [18].
